# Embodying an avatar with a dissimilar appearance enhances insight problem solving

**DOI:** 10.1038/s41598-025-31146-3

**Published:** 2025-12-08

**Authors:** Shih-Yu Lo, Li-Jung Hsu

**Affiliations:** Institute of Communication Studies, National Yang Ming Chiao Tung University, Hsinchu, Taiwan

**Keywords:** Virtual reality, Creativity, Proteus effect, Construal level theory, Psychological distance, Mathematics and computing, Psychology, Psychology

## Abstract

Research on construal level theory suggests that psychological distance enhances creativity, as people tend to be more creative when considering events or objects that are spatially or temporally distant. With immersive technologies, individuals can use different forms of digital representations (or avatars) for work or entertainment in immersive virtual reality (IVR). We hypothesized that embodying an avatar with a dissimilar appearance, compared to a similar one, would induce psychological distance and thus enhance creative thinking and insight problem solving. To test this hypothesis, participants wore an IVR headset and completed creativity and problem-solving tasks while embodying avatars of varying similarity to themselves. The results showed that participants who embodied a dissimilar avatar solved more insight problems than those with a similar avatar. Theoretically, our findings provide further evidence for construal level theory. Practically, our findings suggest immersive technology can be applied to enhance human performance in insight problem solving tasks.

## Introduction

### Creativity

Creativity has long been considered a vital component of human intelligence. Mainstream theories of creativity often define it as a form of divergent thinking— the ability to generate multiple potential solutions^1,2^. However, while divergent thinking is essential, it does not represent all that creativity can be; a creative idea should not only be original, it should also be useful^3^. Thus, originality and usefulness have been recognized as two major criteria of creativity, with roots tracing back from Guilford^2^ to Stein^4^, Hennessey and Amabile^5^, Simonton^6^, and Runco & Jaeger’s “the Standard Definition of Creativity"^7^.

Emotions and movements have been identified as factors that can influence creative performance. For instance, Isen et al^8^. found that positive emotions induced by external stimuli (e.g., comedies or snacks) can lead to higher levels of creativity. Furthermore, Friedman and Förster^9,10^ found that different forms of body movements, such as arm flexion or arm extension, can prompt different degrees of insightful thinking and creativity.

### Creativity and psychological distance

Among the various external factors that can influence creativity, psychological distance plays a particularly important role. Psychological distance refers to an individual’s perceived separation from an event in space, time, hypothetical scenarios, or social relations. Research has shown that greater psychological distance is associated with higher creative performance. For example, Förster et al^11^. found that participants who envisioned their lives one year later, as opposed to those who envisioned their lives the next day, performed better in creative tasks, highlighting the effect of temporal distance. Similarly, Jie et al^12^. demonstrated that participants generated more creative responses when a task was described as originating from a distant location rather than a near one, emphasizing the role of spatial distance.

The link between psychological distance and creativity can be explained through construal level theory^13,14^. This theory suggests that individuals interpret events or objects at different levels of abstraction. Low levels of construal focus on concrete details and specific methods used to accomplish a task, while high levels of construal emphasize abstract aspects such as the purpose or meaning behind a task. For example, the action of “painting a room” can trigger different levels of construal^15^. Low levels of construal might entail “using the paint and brush,” whereas high levels could involve “feeling refreshed.”

Psychological distance is a key factor influencing construal levels^16^. Greater psychological distance leads to higher levels of construal, encouraging individuals to focus on the desirability of achieving a goal without overemphasizing feasibility. This shift in perspective allows for more novel and creative solutions. Conversely, shorter psychological distance results in lower levels of construal, where individuals prioritize feasibility and rely on familiar, previously used methods, thereby limiting creative potential. This explains why increasing temporal distance^11^ or spatial distance^12^ enhances creative thinking.

In summary, psychological distance promotes creativity by encouraging abstract thinking through higher construal levels. When individuals adopt a broader perspective, they are better able to generate innovative solutions rather than focus solely on practical constraints.

### Using IVR to facilitate creativity

With the advancements of technologies that provide immersive virtual reality (IVR), psychological distance can now be artificially induced. IVR refers to an artificial reality created using digital devices that causes users to feel as if they are in a different space^17–19^. The IVR experience is conventionally mediated by a headset that provides users with a rich amount of three-dimensional audio-visual stimuli. In IVR, users can interact with other users who are physically located far away, and they can even embody themselves in different forms or avatars^20^.

Thus, the development of IVR has given rise to a new line of research that investigates how different forms of avatars shape cognitive processes. Inspired by self-perception theory, Yee and Bailenson^21^ introduced the concept of the *Proteus effect*, which refers to the phenomenon whereby the appearance of the avatar affects the user’s attitudes and behaviours. This phenomenon is caused by people inferring their internal states from external cues^22^. For example, studies have found that the body type of the avatar influences the user’s exercise performance^23–26^. More specifically, when embodying an avatar with a taller or larger body shape, people exhibited a higher level of aggression while in the virtual environment, and this benefit was maintained during the subsequent face-to-face negotiation task^27^. In the educational context, students who embodied male avatars scored higher on math tasks (^28–32^). Moreover, in the study by Buisine et al^33^., the participants embodied an avatar that was either a public transport user (an elderly person, a child, a young mother, etc.) or an inventor (a person who wore a lab coat). Their results showed that embodying the former type of avatar led to more user-centred ideas, whereas embodying the latter prompted more techno-centred ideas.

IVR technology has been shown to potentially enhance creative thinking. For example, Guegan et al^34^. found that when people embodied an avatar with the appearance of an inventor during a brainstorming meeting (rather than one with a non-inventor appearance), they considered themselves to be more creative and were able to generate more unique ideas. Furthermore, Van Hooijdonk et al^35^. found that being immersed in an unusual IVR context (e.g., a clothing store) that was incongruent with the task at hand (e.g., naming as many uses of a book as possible) when compared with being a more typical context (e.g., library) led to better performance in terms of creative thinking. A possible explanation for this finding is that congruent environments facilitate retrievals of past experiences, which could potentially cause mental fixation^36^, thereby reducing cognitive flexibility. Incongruent conditions, however, prevent mental fixation and thus lead to better performance in relation to creative thinking.

In addition to enabling users to embody an inventor, providing a context incongruent with the task, we hypothesize that IVR can increase psychological distance to enhance creativity. Previous studies have shown that increasing spatial^12^ or temporal distance^11^ can foster creative thinking, which covers two dimensions of psychological distance that can increase people’s levels of construal^16^. One relevant dimension yet to be explored is social distance. According to construal level theory, an event that involves people who are socially distant can induce higher levels of construal, which may potentially promote creative thinking. However, no prior study has tested this possibility. Therefore, we designed an experiment to test this.

In the present study, social distance is defined as the perceived dissimilarity between individuals. Liviatan et al^37^. demonstrated that participants who shared a common name, initials, or, experiences (e.g., taking the same course) perceived shorter social distance between themselves and others. Their findings suggest that interpersonal similarity is a key determinant of social distance. When individuals perceive others to be similar to themselves, they experience a stronger sense of social proximity. Based on this, the present study manipulated appearance-based similarity to examine whether social distance influences creative thinking.

### Creativity and insight problem solving

In addition to creativity, we also explore another type of psychological mechanism: Insight problem solving. “Insight” indicates the sudden realization of a problem’s solution^1^. There is no standard way to solve insight problems, so finding solutions usually involves people reinterpreting the problems in different ways. People who solve insight problems typically experience an “Aha!” moment—that is, at one point, they do not know the answer, but the next moment, they suddenly discover it^38,39^. As restructuring or reinterpreting the problem is a very important step in insight problem solving^40,41^, the avoidance of mental fixation is essential.

Although creativity and insight problem solving initially appear different, they both engage similar cognitive processes associated with generating novel and useful ideas. Unlike reasoning and decision making, which require controlled, analytic, and conscious processes, both creative thinking and insight problem solving rely heavily on unconscious and associative processes^40^. From a historical perspective, early Gestaltists proposed that both creative thinking and insights follow a four-stage sequence: preparation, incubation, illumination, and verification^42,43^. Individuals begin with preparation, actively working to solve a problem, followed by incubation, where the problem is put aside. After some time, they experience illumination, in which the solution suddenly emerges. Finally, during verification, they express and refine their solution through writing or verbalization. More recently, Ohlsson^44^ reformulates the four-step Gestalt models of creativity as the insight sequence. Empirical studies also support the link between insight problem solving and creative thinking. For instance, performance on insight problem solving correlates with diverging thinking fluency^45^, which predicts creative achievement^46^. Based on the assumption that creative thinking and insight problem solving reflect the same underlying psychological processes, Jia et al^12^. used performance on insight problem solving as an indicator of creative thinking. As a result, the present study not only examined creativity, but also explored insight problem solving as an essential cognitive process.

### Goal of the present study

The emergence of immersive technology allows users to embody different forms while performing tasks. The present study aims to investigate the impact of this emerging technology on human cognition. Specifically, drawing on construal level theory, we hypothesize that using a dissimilar avatar evokes greater perceived social distance, thereby enhancing creativity. Given creativity and insight problem solving share common characteristics—both require uncontrolled and unscripted processes—we further hypothesize that this effect should extend to insight problem solving. To test this hypothesis, we used IVR, which enables users to embody different forms, to examine the effect of avatar similarity on creativity and insight problem solving.

## Methods

An experiment was conducted to test whether embodying an avatar with a dissimilar appearance promoted creative thinking when compared with embodying an avatar with a similar appearance. The avatar embodiment was induced by exposing the participants to visual stimulation consistent with their movements via a head-mounted IVR display while they were performing tasks involving creative thinking and insight problem solving.

### Participants

The experimental protocol for the present study was reviewed and approved by the Institutional Review Board B of National Yang Ming Chiao Tung University, and all methods were performed in accordance with the relevant guidelines and regulations laid down by the ethics committee. All the participants provided informed consent prior to participating in the experiment. To estimate the required sample size, we used the study by Guegan et al^34^. as a reference, where the effect size of an avatar’s appearance on creativity performance *η*^*2*^ was 0.16. We then used G*Power 3^47^ to calculate the sample size, with a sample size of 44 estimated to achieve a power of 80%. Thus, we recruited 44 participants (23 females, *M*_*age*_ = 24.7 years), through advertisements posted on the announcement pages of Facebook groups primarily used by university students. The average number of years participants have used IVR was 0.6 years (13 participants had never used IVR before). All the participants completed the experiment, and were given NTD 200 as compensation after the experiment.

### Materials

The experimental stimuli were presented via Oculus Quest 2 VR head-mounted display. This device can track the user’s head and body movements and adjust their visual field accordingly. The participants were randomly assigned to either the similar-avatar group or the dissimilar-avatar group, with 22 participants in each group. Prior to the experimental day, each participant was required to provide a picture of themselves to allow us to create a preliminary avatar for them. We used the “Edit Avatar” function in Oculus Quest 2 to create the avatar, which enabled us to change the avatar’s body type, skin tone, facial features, etc. We intentionally adjusted these features to either match (for the similar-avatar group) or mismatch (for the dissimilar-avatar group) the participant’s overall appearance, including their gender. On the experimental day, we made minor adjustments to the avatar’s clothing to match (similar-avatar group) or not match (dissimilar-avatar group) what the participant was wearing. Due to potential copyright concerns, the exact avatars used in the experiment are not included in the manuscript; however, they were cartoon-style characters.

We used Horizon Workrooms to create the experimental scenario. It is an application designed to facilitate remote meetings and collaboration by providing users with virtual meeting rooms, virtual whiteboards, and other virtual facilities to hold meetings in an IVR environment. We arranged a mirror in Horizon Workrooms so that the participants were able to see their avatars while performing the experimental tasks.

### Procedure

After entering Horizon Workrooms, each participant could see their avatar in the mirror, and they were encouraged to tilt their head, raise their arms, and move their bodies to see whether their avatar moved with them. The participants were then required to answer a few questions designed to record their basic demographic variables, IVR experience, and their pre-task expectancy concerning the upcoming tasks^12^. The demographic questions included their gender and birth year. The question about IVR experience was *“How long have you been using virtual reality (years)?”* The pre-task expectancy questions were answered using a 7-point Likert scale, including: (1) *How do you feel right now?* (1 = very bad, 7 = very good); (2) *How well will you perform on the following task?* (1 = very badly; 7 = very well); (3) *How much would you like to solve the following task right now* (1 = not at all, 7 = very much); (4) *How important do you feel it is for you to perform well in the following tasks?* (1 = not important at all, 7 = very important). The questionnaire was presented on the Qualtrics platform. We projected the desktop view of Qualtrics into the IVR environment so that the participants could fill out the questionnaire inside the virtual environment.

After completing the first part of the questionnaire, the participants performed the idea-generation task, as the measure of creativity. We used the same questions as those used in the study by Guegan et al^34^., where the participants were required to “imagine a crazy solution for traveling on snow, sand, or water” as Question A and to “imagine a silent flying mode of public transportation for the future” as Question B. They were informed that there were no right or wrong answers and that the examples they generated could be commonplace or creative. They provided responses by writing down as many examples as they could think of on virtual sticky notes in the IVR environment, which corresponded to a digital writing board in the physical environment. The participants wrote down their ideas on a digital writing board with a controller. In the IVR, this appeared as writing their answers on sticky notes.

The second task entailed insight problem solving. We used the same questions as those used in the study by Jia et al^12^.:

#### Problem 1

A prisoner was attempting to escape from a tower. He found a rope in his cell that was half as long enough to permit him to reach the ground safely. He divided the rope in half, tied the two parts together, and escaped. How could he have done this?

#### Solution

He unravelled the rope lengthwise and tied the remaining strands together.

#### Problem 2

A dealer in antique coins got an offer to buy a beautiful bronze coin. The coin had an emperor’s head on one side and the date 544 B.C. stamped on the other. The dealer examined the coin, but instead of buying it, he called the police. Why?

#### Solution

In 544 B.C., Jesus had not been born, so a coin from that time would not be marked “B.C.”

#### Problem 3

**Fig. 1:**
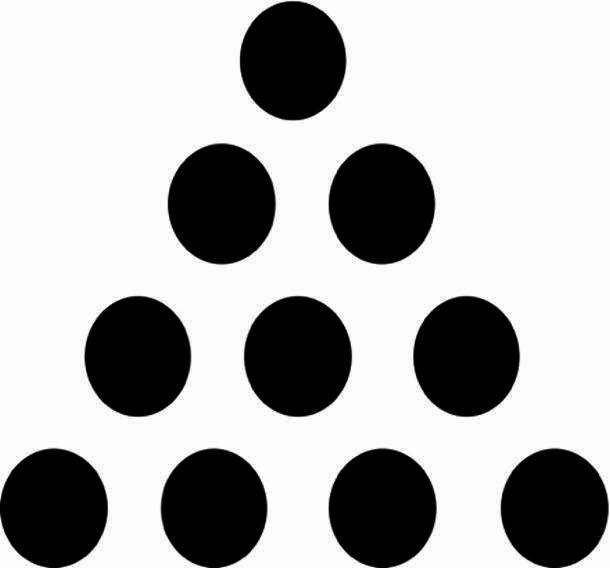
Problem 3 from the insight problem solving task.

Show how you can make the triangle below point downward by moving only three of the circles (Fig. 1).

#### Solution

**Fig. 2:**
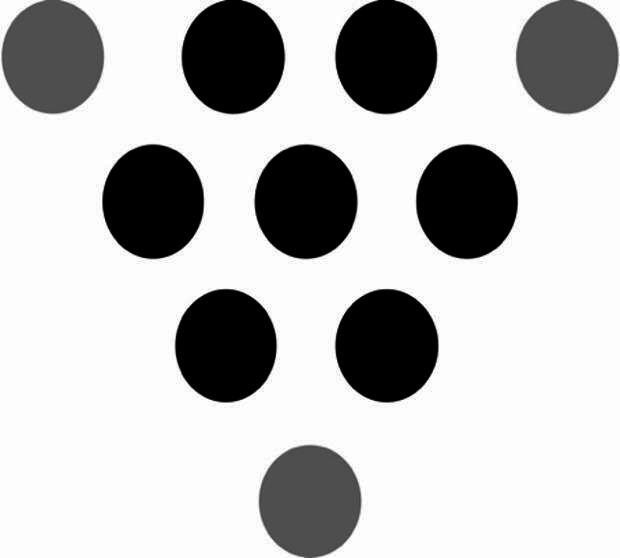
The solution to Problem 3. Simply move the top, bottom-left, and bottom-right circles shown in Fig. 1 to the locations marked with grey circles.

Please refer to Fig. 2.

The participants had to answer each question within two minutes. After completing all the tasks, the participants filled out the post-task questionnaire.

The first part of the post-task questionnaire measured avatar embodiment^18^ using a 7-point Likert scale (1 = strongly disagree; 7 = strongly agree). The items were: 1*) While interacting in VR*,* you had the feeling of being in the avatar’s skin.* (2) *While interacting in VR*,* you sometimes completely forgot about yourself because you were so focused on the avatar’s actions.* (3) *While interacting in VR*,* you had the feeling of actually being the avatar.*

Following the avatar embodiment measure was the post-task reflection^12^ measure, which included the same five items used in Jia et al. (2009), with one additional item, *“how do you feel now?*” All the items were rated on a 7-point Likert scale. The items were: (1) *How do you feel now* (1 = very bad, 7 = very good)? (2) *Was it interesting to generate ideas and solve insight problems* (1 = surely no; 7 = surely yes)? (3) *If asked to solve other problems*,* would you be happy to do it* (1 = surely no; 7 = surely yes)? (4) *Did you find it fun to generate ideas and solve insight problems* (1 = surely no; 7 = surely yes)? (5) *Did you find it hard to get really involved with generating ideas and solving insight problems* (reverse coding; 1 = surely no; 7 = surely yes)? (6) *Did you thoroughly enjoy generating ideas and solving insight problems* (1 = surely no; 7 = surely yes)?

Next, perceived avatar similarity^37^ was measured as a manipulation check to assess whether our similarity manipulation indeed produced different levels of perceived avatar similarity in the similar-avatar and dissimilar-avatar groups. Participants responded to one item on a 7-point Likert scale: *How similar do you think the avatar was to you?* (1 = not similar at all, 7 = very similar)?

Perceived social distance was then measured using the Inclusion of Other in the Self (IOS) Scale^48^. Participants were shown seven pairs of circles with varying degrees of overlap, with one representing the self and the other representing others. The degree of overlap indicated how close they perceived their avatar to be to themselves. The entire experiment took approximately 1 h to complete.

### Analysis

The *fluency* and *uniqueness* indices were used to evaluate the participants’ performance in idea-generation task. The fluency index refers to the number of ideas the participants generated. For *uniqueness*, we used a method similar to the study of Guegan et al. (2016) to define uniqueness. Uniqueness, similar to the study of Guegan et al. (2016), was defined by the number of responses that are different from others. Specifically, we firstly collapsed all the repeated or synonymous responses in the two corpora for Questions A and B, then asked a human judge to group conceptually similar responses into the same category. Uniqueness is defined by the number of responses that did not belong to a category with another response. Unlike Guegan et al. (2016) who asked two human judges to classify the responses, we used an artificial intelligence agent, a Chat Generative Pre-trained Transformer (ChatGPT), to perform this task.

The ChatGPT is language model developed by OpenAI. For the present study, the GPT-3.5 was used. The prompt was: “If the task were to imagine a crazy solution for traveling on snow, sand or water (or to imagine a silent flying mode of public transportation for the future), please put semantically related responses below into the same category.”

After obtaining fluency and uniqueness as two indices for creativity, and numbers of insight problems solved as the index of insight problem solving, we used these indices as dependent variables, and avatar similarity as the independent variable to perform a one-way analysis of variance (ANOVA). More detailed analysis methods will be explained in the Results section.

### Manipulation check

To ensure that our manipulation of avatar similarity was valid, we employed the same method as that used by Liviatan et al^37^.—that is, asking the participants how similar they felt their avatar was to themselves. The independent samples t-test showed that the rating in the similar-avatar group (*M* = 5.59; *SD* = 0.50) was significantly higher (*t*(31.01) = 14.87, degree of freedom was adjusted as the homogeneity assumption was violated, *p* <.01, *d* = 4.48) than that in the dissimilar-avatar group (*M* = 2.05; *SD* = 1.00). Therefore, our manipulation was successful. Moreover, our manipulation of avatar similarity successfully induced different degrees of social distance, as the IOS score for the participants in the similar-avatar group (*M* = 5; *SD* = 1.27) was higher (*t*(42) = 5.71, *p* <.001, *d* = 1.72) than that for the participants in the dissimilar-avatar group (*M* = 2.55; *SD* = 1.57). The correlation between perceived similarity and social distance was high (*r* =.68, *t*(42) = 5.98, *p* <.001). These results suggest that perceived similarity strongly predicts social distance.

## Results

### Reliability

In terms of the measurements involving multiple items, the Cronbach’s alpha value was 0.66 for pre-task expectancy, 0.83 for avatar embodiment, and 0.84 for post-task reflection. We dropped one item related to pre-task expectancy to achieve higher reliability (*How important do you feel it is for you to perform well in the following tasks?*), and the updated Cronbach’s α value was 0.71. Further analysis of the participants’ pre-task expectancy was based on the remaining three items. For all constructs measured using multiple items, the mean value was used as the index of each construct.

### Idea generation: fluency

**Fig. 3:**
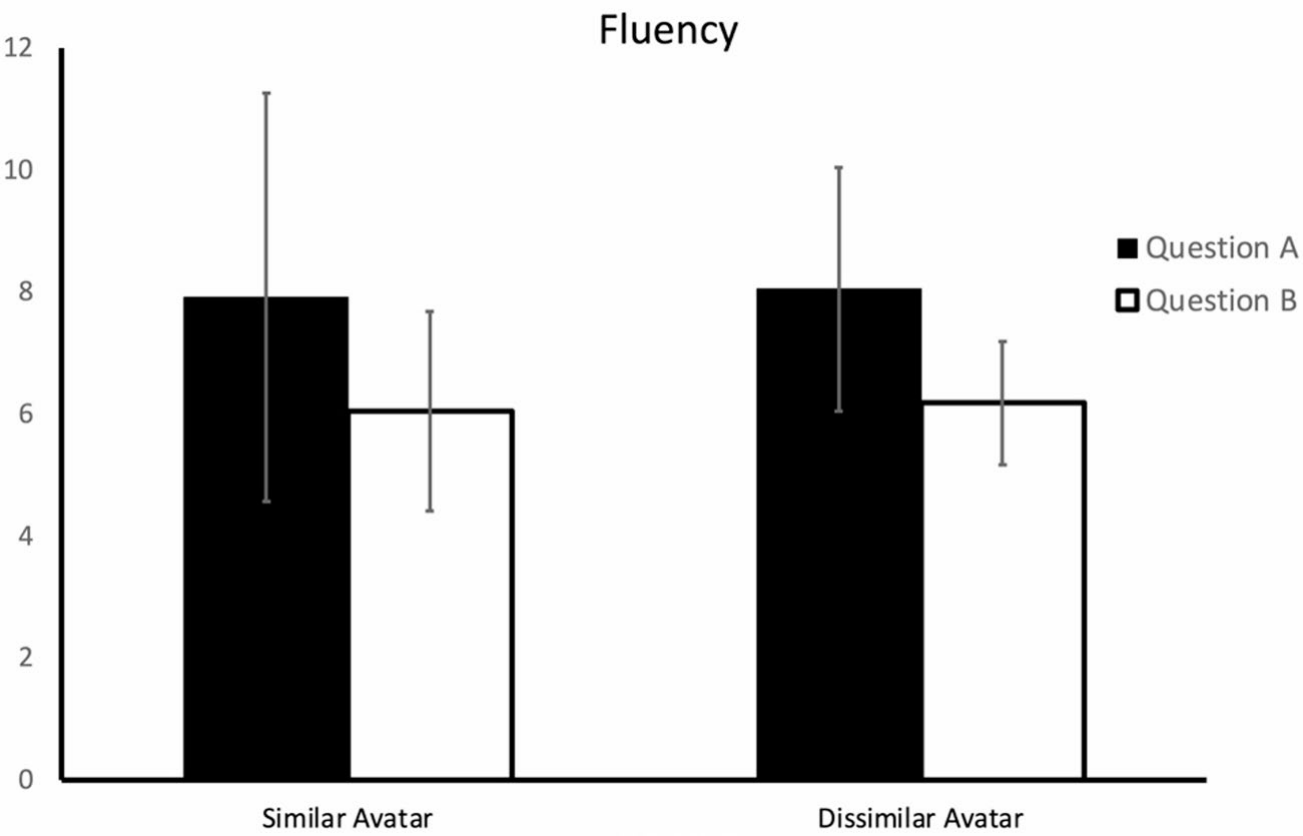
Degree of fluency of the participants’ responses to the two questions in the two experimental groups. The error bars indicate 95% confidence intervals.

The fluency index was then subjected to a one-way analysis of variance (ANOVA), with the independent variable being “avatar similarity.” Fluency did not significantly differ (*F*(1,42) = 0.005; *p* =.94; *η*^*2*^ < 0.001) between the similar-avatar group (*M* = 7.91; *SD* = 7.55) and the dissimilar-avatar group (*M* = 8.05; SD = 4.50) for Question A (*solutions for traveling on snow*,* sand*,* or water*). Moreover, for Question B (*silent flying mode of public transportation*), the difference between the similar-avatar group (*M* = 6.05; SD = 3.67) and the dissimilar-avatar group (*M* = 6.18; *SD* = 2.28) again did not reach statistical significance (*F*(1,42) = 0.02; *p* =.88; *η*^*2*^ = 0.001) (Fig. 3).

### Idea generation: uniqueness

The participants generated 351 responses for Question A and 269 responses for Question B. After collapsing all the repeated or synonymous responses in the two corpora for Questions A and B, we obtained  245 unrepeated responses for Question A and 193 for Question B. We then asked a human judge and a ChatGPT judge to group conceptually similar responses into the same category. Uniqueness is defined by the number of responses that did not belong to a category with another response. The inter-rater reliability between the human and the ChatGPT judges was indicated by the correlation coefficients between the values of uniqueness derived from the two judges, and they were 80% for Question A and 64% for Question B. Table 1 presents some examples of the ideas that were considered unique for both judges.

**Table 1:** Examples of the participants’ responses during the idea-generation task.

Solutions for traveling on snow, sand, or water (Question A)	Silent flying mode of public transportation (Question B)
• Ninja tools strapped to the feet	• Making headgear with the earmuffs closed and attaching wings to it.
• Using Elsa’s magic to freeze the water.	• Building a magnetic metro system in the air.
• Tying a balloon to the body.	• Organizing a stork delivery team to form a passenger transport company.
• Wearing Iron Man’s equipment.	• Riding a unicorn that does not produce any sound.
• Inventing high-tech mechanical legs.	• Making shoes that can float in the air.
• Turning into an animal and walking past it.	• Using many drones to form a flying platform.
• Being blown away by the wind.	• Just removing the people who feel noisy.
• Putting on shoes made of a non-Newtonian fluid.	• Using a downsizing light to reduce body size, sitting on a paper plane, and then asking a goblin to shoot the plane.

**Fig. 4:**
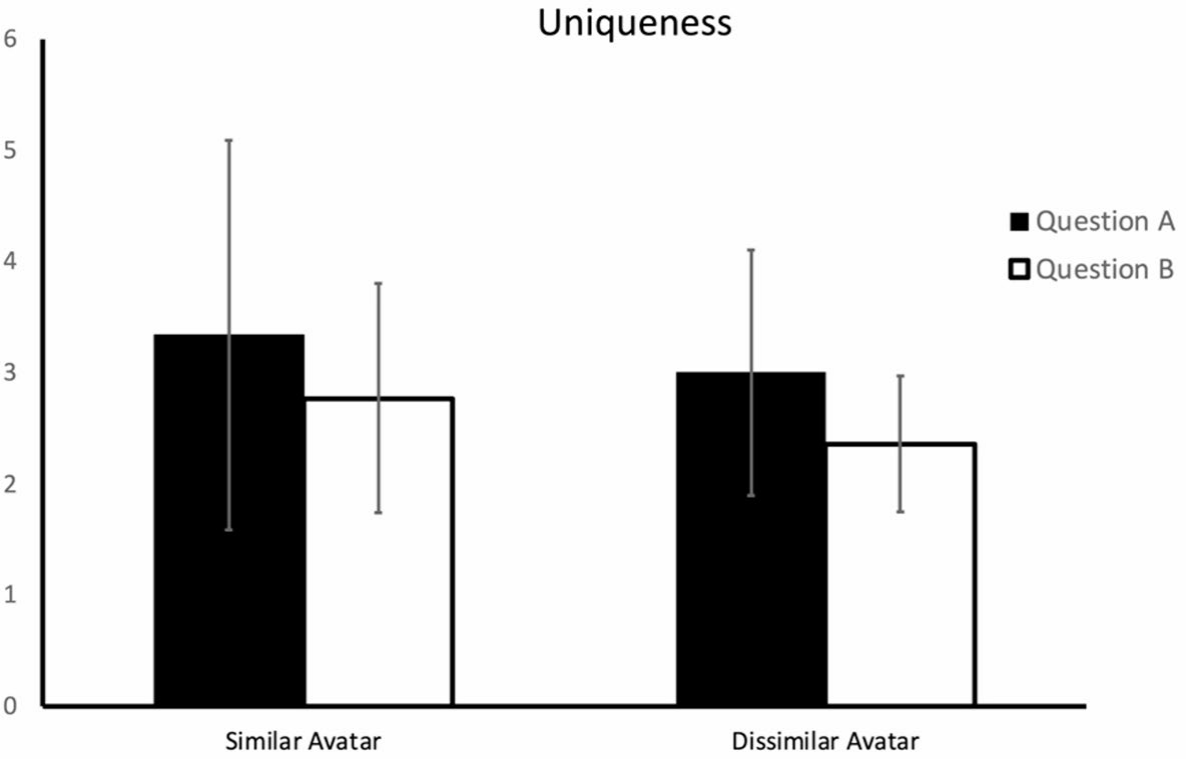
Degree of uniqueness of the participants’ responses to the two questions in the two experimental groups. The error bars indicate 95% confidence intervals.

We then used the averaged values for the human judge and the AI judge as the index of uniqueness. The ANOVA results revealed that the numbers of unique ideas in the similar-avatar group (*M* = 3.34; *SD* = 3.94) and the dissimilar-avatar group (*M* = 3.00; *SD* = 2.49) did not significantly differ (*F*(1,42) = 0.12; *p* =.73; *η*^*2*^ = 0.003) for Question A. In addition, the numbers of unique ideas in the similar-avatar group (M = 2.77; SD = 2.33) and the dissimilar-avatar group (*M* = 2.36; *SD* = 1.39) also did not significantly differ (*F*(1,42) = 0.50; *p* =.48; *η*^*2*^ = 0.01) for Question B (Fig. 4).

### Insight problem solving

**Fig. 5:**
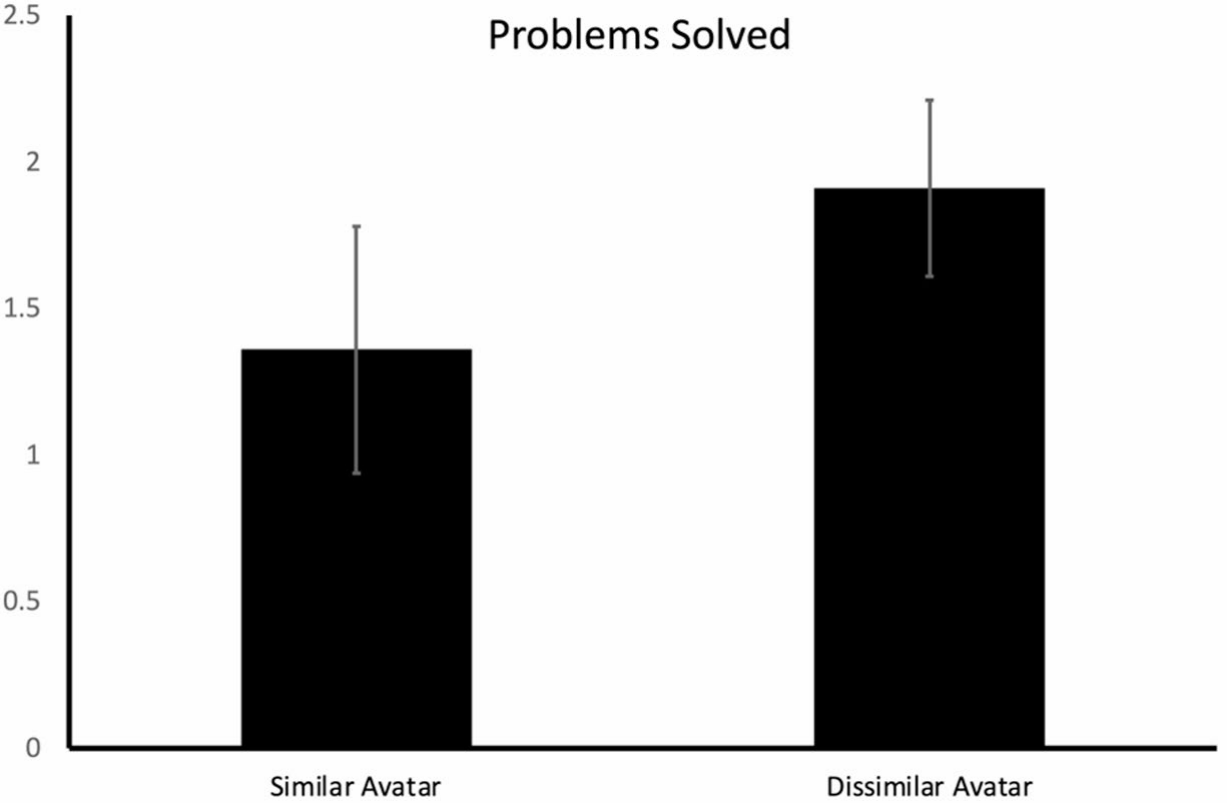
Number of insight problems solved. The error bars indicate 95% confidence intervals.

The participants’ insight problem solving performance was indexed by the number of questions that they successfully solved. According to the ANOVA results, avatar similarity exerted a significant effect (*F*(1, 38.08) = 4.76, degree of freedom was adjusted as the homogeneity assumption was violated; *p* =.04; *η*^*2*^ = 0.10), where the number of solved questions was higher in the dissimilar-avatar group (*M* = 1.91; *SD* = 0.68) than in the similar-avatar group (*M* = 1.36; *SD* = 0.95). Please refer to Fig. 5.

### Examination of possible confounders

In addition to participants’ performance in creativity and insight problem solving, we also recorded participants’ IVR experience, pre-task expectancy, post-task reflection, and avatar embodiment. As these variables might potentially be confounders, we still provide their information here.

For IVR experience, we categorized people with previous IVR experience as the experienced participants, and people with no previous IVR experience as inexperienced participants. Chi-square analysis showed that the proportions of experienced to inexperienced individuals in the similar-avatar and dissimilar-avatar did not significantly differ (χ^2^(1) = 0.11, *p* =.74). Therefore, IVR experience should not be a confounder.

For pre-task expectancy, its value in the similar-avatar group (*M* = 5.83; *SD* = 0.63) was higher (*t*(42) = 2.49; *p* =.02; *d* = 0.75) than in the dissimilar-avatar group (*M* = 5.33; *SD* = 0.70); however, after completing the task, the post-task reflection on the task in the similar-avatar group (*M* = 5.90; *SD* = 0.72) and dissimilar-avatar group (*M* = 5.60; *SD* = 0.80) did not significantly differ (*t*(42) = 1.30; *p* =.20; *d* = 0.39). It should be noted that the measurement of the participants’ pre-task expectancy took place after they had entered the IVR environment and embodied an avatar; therefore, there was a tendency for the participants embodying a similar-looking avatar to experience higher pre-task expectancy than the participants embodying a different-looking one.

As the participants in the similar-avatar group exhibited higher pre-task expectancy than the participants in the dissimilar-avatar group, it is possible that the differing problem-solving performance in the two groups could have been mediated by pre-task expectancy. We tested this using the PROCESS macro^49^. More specifically, we used avatar similarity as the independent variable (1: similar avatar; 2: dissimilar avatar), pre-task expectancy as the mediator, and the number of problems solved as the outcome variable. We used Model 4 (mediation analysis) with 5000 resampling times. The 95% confidence interval for the estimated mediation effect was (−0.15, 0.25), which included 0 and indicated a lack of statistical significance. Thus, although the participants in the similar-avatar group had higher pre-task expectancy than those in the dissimilar-avatar group, the higher expectancy was unlikely to be the cause of their worse problem-solving performance.

For avatar embodiment, its value in the similar-avatar group (*M* = 5.23; *SD* = 1.53) was not significantly different (*t*(42) = 1.58; *p* =.12; *d* = 0.48) from that in the dissimilar-avatar group (*M* = 4.52; *SD* = 1.45). Therefore, avatar embodiment should not be a confounder. This result might come as surprising. A more detailed discussion will be provided in the Discussion section.

## Discussion

### Summary of results

The emergence of IVR has enabled people to embody different digital representations for work and entertainment purposes. One important topic is how the adoption of IVR affects our cognitive processes. Drawing on construal level theory, we hypothesize that embodying a dissimilar avatar, as opposed to a similar one, should increase perceived social distance, which presumably raises construal levels and enhances people’s performance in creative thinking and insight problem solving. Our experimental results were consistent with this hypothesis in terms of insight problem solving: Participants in the dissimilar-avatar group solved more insight problems than those in the similar-avatar group. However, in terms of fluency and uniqueness indices, no significant effect of avatar similarity was observed.

### Creative thinking and insight problem solving

Creativity has long been considered to be associated with novel and useful ideas^2,4–7^. Consistent with the view that creativity is a dynamic process subject to change^50^, levels of construal affect creativity. Higher construal levels foster abstract thinking and prioritize desirability over feasibility. The prioritization of desirability enables people to generate useful ideas that help achieve a certain goal, while the de-emphasis of feasibility encourages people to generate ideas that are not constrained by their life experiences. Therefore, high levels of construal could promote creative thinking.

Creative thinking and insight problem solving both rely on unconscious and associative processes^40^. According to traditional Gestaltists, both involve the sequence of preparation, incubation, illumination, and verification^42,43^. Therefore, it is very likely that external factors fostering creative thinking also enhance insight problem solving. Interestingly, the effect of avatar similarity was only observed in relation to the insight problem solving task and not the creativity task. We speculate that the perceived similarity was not properly manipulated in our creativity task. During the idea-generation task, the participants provided responses by writing down their ideas on virtual sticky notes (corresponding to a digital writing board in the physical environment). While they were writing, they were looking down at the sticky notes and so not paying attention to the avatar image in the virtual mirror. However, the problem-solving task was conducted differently, as the participants provided verbal reports and could still see the avatar in the mirror while solving the problems. It is possible that the visibility of the avatar may be critical for the similarity effect to influence cognitive performance.

### The role of avatar embodiment

A curious finding in this study is the lack of a significant difference in avatar embodiment between the similar-avatar and dissimilar-avatar groups. This result may relate to the concept of homuncular flexibility, emerged in the late 1980’s by Jaron Lanier and colleagues. The cortical homuncular represents the mapping between cortical areas and the corresponding body parts, and this mapping has been shown to be remarkably flexible. For example, Ramachandran and Rogers-Ramachandran^51^ demonstrated that upper limb amputees could experience an illusory limb by viewing the reflection of their uninjured limb in a mirror box. Similarly, Kilteni, Normand, Sanchez-Vives, and Slater^52^found that participants could embody avatars with unrealistically long arms and still experience ownership of them, as evidenced by their defensive reactions when the avatar was threatened. In another study, Won et al.^53^, introduced participants to a virtual third arm in the IVE, which they learned to control through wrist rotation. Although their initial performance was poorer than in the normal-arm condition, their performance improved more rapidly than that in the normal-arm condition. Together, these studies all demonstrate that humans can easily adapt to novel or even unrealistic body forms. Compared with these studies, the avatars used in this present study —whose appearance remained relatively realistic — should not have posed much difficulty for participants to embody.

### Potential influence of gender identity

The goal of this study was to examine how appearance-based similarity can affect performance in creative thinking and insight problem solving. To strengthen the manipulation, participants in the similar- and dissimilar-avatar groups embodied avatars of the same or a different gender, respectively. However, this design choice may have introduced an additional factor beyond visual appearance—namely, gender identity. Prior research has shown that avatar genders^54^ and sexual characteristics^55^ can influence behaviour and cognition, raising the possibility that the observed effect may partly reflect gender related processes rather than purely visual dissimilarity.

We cannot exclude this explanation. However, we consider “appearance” to be a broad construct that includes visual as well as gender-related cues. Even if the key mechanism underlying enhanced insight problem solving performance involves a temporary shift in gender identity, this shift was induced through changes in visual appearance. Thus, it remains consistent with our main conclusion of the present study that “appearance dissimilarity” facilitates insight problem solving. Nevertheless, we recognise that “appearance” is a theoretically broad concept, and future research should aim to disentangle which specific element —such as gender cues or sexual features—constitutes the critical component driving this effect.

### Limitations

This study has a number of limitations. First, we used only a very limited number of measurements—two idea-generation tasks for creativity and three problems for insight problem solving—which means that the results might not be generalizable to other measurements of creativity.

Second, the manipulation of similarity was not applied uniformly across participants. Therefore, it remains unclear which specific element of “similarity” was responsible for the observed effect. Possible contributing factors include facial features, body features, clothing, or gender-related characteristics, as described in the previous section. Future research should examine the role of each of these features more systematically. In the present experiment, some of the adjusted features were relatively stable (e.g., body shape, skin tone, or gender-related features) and might have become internalized as part of “identity- related” processes, whereas others were more transient (e.g., clothing). Determining which types of features play a more dominant role in shaping the insight problem-solving processes represents an important direction for future investigation.

Third, the measurement of creativity may not be entirely reliable, particularly for the uniqueness score in Question B. Moreover, we used ChatGPT to assess uniqueness, which may be affected by the problem of AI hallucination—the tendency of large language models (LLMs) to generate inaccurate or fabricated information. In the case of assessing uniqueness, the potential impact of AI hallucination is similar to that of human raters who fail to follow the rating instructions properly, which underscores the importance of assessing inter-rater reliability. Unfortunately, the inter-rater reliability for Question B was low. Nevertheless, creativity in this study was indexed by both fluency and uniqueness, each measured by two questions. The results revealed a consistent pattern across the four indices (two dimensions x two questions), suggesting that the low reliability of one measure is unlikely to substantially affect our overall conclusion. Still, the main effect of avatar similarity was not significant, and thus, our conclusion should be interpreted with caution.

Lastly, the actual mechanism mediating the avatar-similarity effect might not be clear. We developed our hypothesis based on construal level theory, and the gathered data supported that hypothesis. However, our results could potentially be mediated by other mechanisms. For example, embodying a dissimilar avatar could have promoted creative thinking by reducing mental fixation^35^. Alternatively, the effect observed in the present study could have been a priming effect^56^. In the similar-avatar group, the participants were exposed to an avatar with a similar appearance, and the visual similarity might have primed them to solve problems with similar strategies as those applied in their past experiences, whereas the visual dissimilarity in the dissimilar-avatar group might have primed the participants to think dissimilarly to their usual life experiences.

Another unsolved issue in the present study is whether the effect of avatar similarity originates from “facilitation” of the dissimilar avatar, or the “inhibition” of the similar avatar. Our speculation is that the “dissimilar avatar” condition creates an unusual situation that is rarely experienced in daily life. Therefore, the observed effect is likely driven by the “facilitation” of the dissimilar avatar. Nevertheless, a separate study that includes a baseline condition, in which participants do not see any avatar, is needed to address this issue.

## Contributions

Theoretically, our results further validate the effect of psychological distance on construal levels and highlight the dynamic nature of creativity and insight problem solving. Empirically, while previous studies have demonstrated the impact of psychological distance on creativity or insight problem solving in the spatial^12^ and temporal^11^ dimensions, our study extends the evidence to the social dimension of psychological distance. Practically, the findings of the present study suggest a new perspective on technology. Humans develop technologies primarily to make life easier; however, its influence extends beyond convenience, potentially shaping our cognition. By using technology effectively, we might maximize the potential of our cognitive system — just as avatar appearance can influence our problem-solving capacity.

## Data Availability

The data that support the findings of this study are available on request from the corresponding author.
